# Quantum Material-Based Self-Propelled Microrobots for the Optical “On-the-Fly” Monitoring of DNA

**DOI:** 10.1021/acsami.3c09920

**Published:** 2023-12-11

**Authors:** Jyoti, Jose Muñoz, Martin Pumera

**Affiliations:** † Future Energy and Innovation Laboratory, Central European Institute of Technology, Brno University of Technology (CEITEC-BUT), 61200 Brno, Czech Republic; ‡ Faculty of Electrical Engineering and Computer Science, VSB - Technical University of Ostrava, 17. listopadu 2172/15, 70800 Ostrava, Czech Republic; § Department of Medical Research, China Medical University Hospital, China Medical University, No. 91 Hsueh-Shih Road, Taichung 4040, Taiwan

**Keywords:** microrockets, fluorescence, self-propelled micromotors, DNA biosensor, FRET

## Abstract

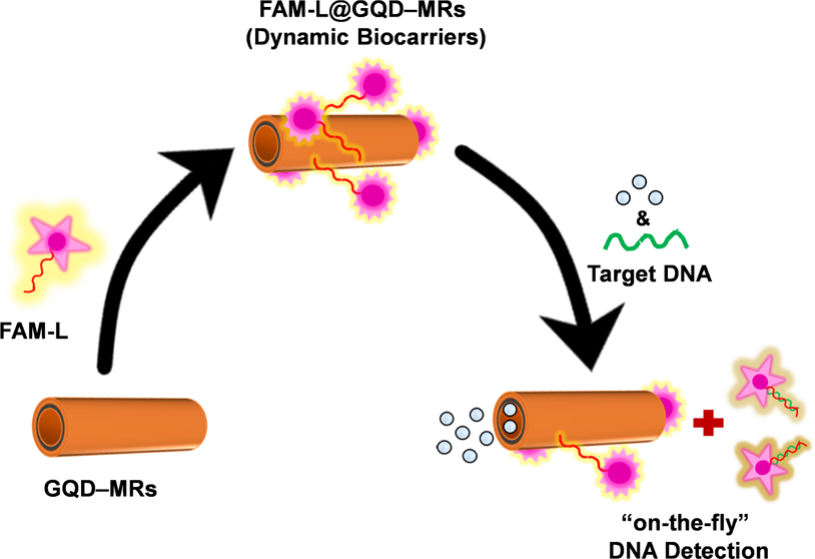

Quantum dot-based materials have been found to be excellent platforms for biosensing and bioimaging applications. Herein, self-propelled microrobots made of graphene quantum dots (GQD–MRs) have been synthesized and explored as unconventional dynamic biocarriers toward the optical “on-the-fly” monitoring of DNA. As a first demonstration of applicability, GQD–MRs have been first biofunctionalized with a DNA biomarker (i.e., fluorescein amidite-labeled, FAM-L) via hydrophobic π-stacking interactions and subsequently exposed toward different concentrations of a DNA target. The biomarker–target hybridization process leads to a biomarker release from the GQD–MR surface, resulting in a linear alteration in the fluorescence intensity of the dynamic biocarrier at the nM range (1–100 nM, *R*^2^ = 0.99), also demonstrating excellent selectivity and sensitivity, with a detection limit as low as 0.05 nM. Consequently, the developed dynamic biocarriers, which combine the appealing features of GQDs (e.g., water solubility, fluorescent activity, and supramolecular π-stacking interactions) with the autonomous mobility of MRs, present themselves as potential autonomous micromachines to be exploited as highly efficient and sensitive “on-the-fly” biosensing systems. This method is general and can be simply customized by tailoring the biomarker anchored to the GQD–MR’s surface.

## 1 Introduction

In recent years, self-propelled microrobots (MRs)^1−4^—which can exhibit autonomous motion by harnessing chemical energy—have attracted great attention in different fields, including catalysis, environmental remediation, cancer therapy, and protein detection, among others.^5−10^ In particular, self-propelled MRs are currently at the forefront of analytical chemistry owing to their unique capability to perform “on-the-fly” biorecognition. The term “on-the-fly” refers to the capability of MRs to perform chemical preconcentration or interactions with target analytes while in motion.^11,12^ The main benefits of chemistry “on-the-fly” rely on the suitability of MRs to rapidly preconcentrate targets on their surfaces (even in small volumes of complex biosamples), accelerating interactions while avoiding several samples post-treatments (e.g., washing/mixing procedures).^3,13−15^

Graphene quantum dots (GQDs) are a type of 0D carbon nanoallotrope that present excellent fluorescent features for optical analyses.^16,17^ In addition, the high solubility, low toxicity, and excellent water solubility of GQDs, together with their sp^2^-like skeleton, make them ideal for processing carrier tasks in aqueous and/or physiological environments.^18−21^ In particular, the sp^2^-like skeleton of GQDs can behave as fluorescence resonance energy transfer (FRET) acceptors or donors by noncovalently absorbing (e.g., π-stacking interactions or hydrophobic interactions) biomaterials, like single-strand DNA (ssDNA).^22,23^ Beyond their aforementioned benefits, the exploration of GQDs as a material for MR fabrication is almost an unexplored field, and Escarpa’s group is leading this field. In particular, they have demonstrated the advantages of using graphene quantum dots-based self-propelled microrobots (GQD–MRs) by means of rich surface chemistry and high surface area, favoring sensing activity.^24,25^

Detection methods involving optical readouts represent a pivotal strategy to evaluate DNA hybridization between a specific nucleic acid target and a complementary nucleic acid probe.^26−29^ Optical DNA biosensing is primarily based on the FRET principle, which relies on the resonance energy transfer from an excited donor fluorophore to a corresponding acceptor fluorophore.^30^ This donor–acceptor relationship helps to improve the analytical performance of the biosensor effectively, resulting in a highly sensitive technique.^31^ Otherwise, the water solubility of GQDs is known to facilitate homogeneous assays, which are vital to DNA detection.^32−34^ The driving force of any DNA hybridization process relies on an adequate close proximity of the complementary strands of DNA for proper interactions, in which diffusion and transport are the main kinetic limiting steps.^35^ In order to overcome this drawback, microrobots have provided new insights into the field of analytical chemistry by improving fluid mixing and localized convection. Compared to conventional methods, the implementation of microrobots can dramatically improve probe interaction for lower samples and reagent usage without the implementation of an external mixing source, the fact that can improve kinetic processes by reducing incubation times by “on-the-fly” reactions.^11,36−39^ Although few types of self-propelled MRs have already been proposed as DNA biosensing platforms employing different readouts,^39−42^ to the best of our knowledge, the exploration of GQD–MRs for this aim is nowadays an unexplored field.

**Scheme 1:**
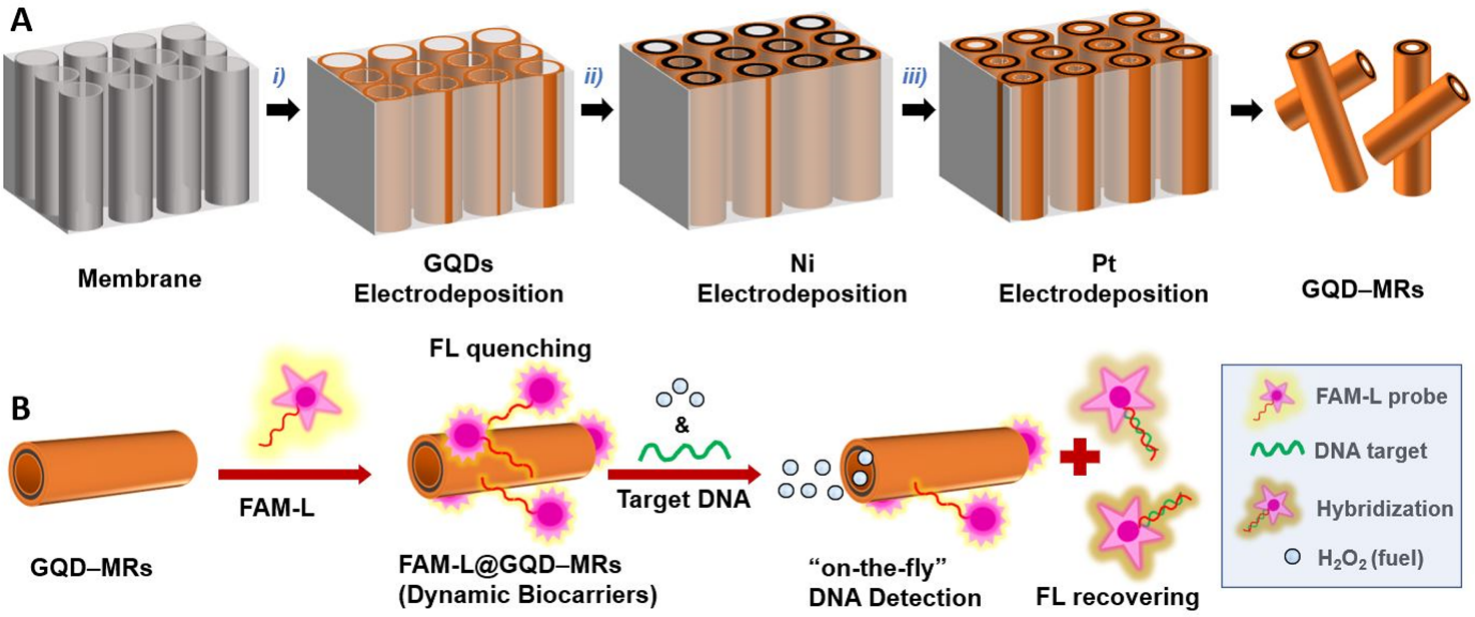
Fabrication of Self-Propelled GQD–MRs and Their Exploitation as Dynamic Biocarriers for the Optical “On-the-Fly” DNA Determination (A) GQD–MRs were synthesized via membrane-assisted electrodeposition of (i) GQDs (outer layer), (ii) Ni (middle layer), and (iii) Pt (inner layer). (B) Biofunctionalization of GQD–MRs with a biomarker (i.e., FAM-L probe) via π-stacking interactions, where the resulting FAM-L@GQD–MRs (dynamic biocarriers) promote a quenching in the fluorescence activity of the probe. The optical analytical assay relies on “On-the-Fly” DNA determination using different concentrations of a complementary DNA target under fuel-induced motion (1% v/v H_2_O_2_), where the DNA hybridization process between the probe and the target derives in an FL recovery.

Herein, dynamic biocarriers made of GQD–MRs have been synthesized and evaluated toward the “on-the-fly” determination of DNA. For this aim, tubular GQD–MRs were fabricated via a membrane-assisted electrodeposition method^43−45^ by electrochemically depositing GQDs (chemically active surface, outer layer), Ni (magnetic core, middle layer), and Pt (motion inducer, inner layer); see Scheme 1A for illustration. The inner walls of GQD–MRs with deposited platinum were responsible for inducing motion by the catalytic disproportionation of H_2_O_2_ into H_2_O and O_2_.^46^ The formation of the O_2_ molecules triggers a nucleation process, leading to the subsequent growth of oxygen bubbles. These bubbles can then diffuse and ultimately pop out from an open end of the asymmetric tubular form of GQD–MRs. As a result, when a bubble is expelled from one end of the tube, a movement takes place in the opposite direction.^46−48^ Afterward, the resulting GQD–MRs were biofunctionalized with a biomarker probe (i.e., fluorescein amidite-labeled, FAM-L). According to the FRET phenomena, the FAM-L probe acted as a fluorophore, while the GQD–MRs served as the fluorescence quenching platform. The noncovalent π–π stacking interactions between (i) the structures of the nucleobases of the FAM-L probe and (ii) the sp^2^-rich skeleton of GQDs leads to the adsorption of the FAM-L probe on GQD–MRs,^22^ resulting in the FAM-L@GQD–MR dynamic biocarriers. Such π–π stacking interactions are the ones responsible for fluorescence quenching. Nonetheless, it is important to note that the interactions between the FAM-L and GQD–MRs involve a continuous competition between electrostatic repulsion and hydrophobic interactions.^49^ For the optical analytical assay, the resulting dynamic biocarriers were propel-induced by utilizing a 1% v/v H_2_O_2_ in buffered medium containing different concentrations of a complementary DNA sequence (DNA target). The changes in the fluorescence (FL) emission of the FAM-L probe with increasing concentrations of DNA target—owing to the hybridization process (inputs)—were used as the optical output signals (see Scheme 1B).^23^ Further, the selectivity of the devised dynamic biocarriers was also interrogated by using both mismatch and noncomplementary DNA sequences.

## 2 Experimental Studies

### 2.1 Materials, Chemicals, and DNA Sequences

GQDs, H_2_O_2_, and sodium dodecyl sulfate (SDS) were provided by Sigma-Aldrich. Commercial Ni and Pt plating solutions were purchased from Singapore. DNA sequences were obtained from Sigma-Aldrich (Czech Republic), which are given as follows: FAM-L DNA probe: 5′ [6FAM] ACC AGG CGG CCG CAC ACG TCC TCC AT 3′; DNA target: 5′ATG GAG GAC GTG TGC GGC CGC CTG GT 3′; mismatch DNA: 5′ ATG GAG GAC GTG CGC GGC CGC CTG GT 3′; noncomplementary DNA target: 5′ A-AAA GTG TTT TTC ATA AAC CCA TTA TCC AGG ACT GTT TAT AGC TGT TGG AAG GAC TAG GTC 3′. Biological fluids (i.e., sigmatrix urine diluent (mimics human urine) and plasma from humans) for the implementation experiments were obtained from Sigma-Aldrich.

### 2.2 Synthesis of Self-Propelled GQD–MRs

GQD–MRs were prepared by using our established membrane-assisted electrodeposition method (see Scheme 1A).^44,45,50^ Briefly, a 100 nm thick Au layer was sputtered on a Whatman Cyclopore polycarbonate membrane (3 μm pore size) via electron-beam evaporation and subsequently affixed on a piece of Cu tape as the electrical contact to fabricate a working electrode. Then, it was placed in a three-electrode configuration cell using a Pt wire and an Ag/AgCl (1 M KCl) electrode as the counter and reference electrodes, respectively. Electrochemical depositions were run in an AUTOLAB potentiostat (Metrohm). First, (i) the outer layer was made by depositing GQDs employing a 0.1 mg·mL^–1^ dispersion (support electrolyte: 0.1 M H_2_SO_4_ containing 0.5 M Na_2_SO_4_) via cyclic voltammetry (CV): potential window: +0.3 to −1.5 V vs Ag/AgCl; scan rate: 50 mV·s^–1^; number of cycles: 40 cycles. Afterward, (ii) Ni middle layer deposition was carried out by chronoamperometry (bias potential: +1 V vs Ag/AgCl; time: 60 s), while chronopotentiometry (current: −20 mA; time: 500 s) was utilized for (iii) the Pt inner layer electrodeposition. Once the electrodeposition was done, the membrane was detached from the copper tape, carefully washed with deionized water, and hand-polished with an alumina slurry (0.5 μm) in order to remove the Au layer. Then, the membrane was thoroughly washed (3 times) with deionized, dissolved in dichloromethane, and finally washed with isopropanol, ethanol, and deionized water thrice under ultrasound. Finally, the resulting self-propelled GQD–MRs were magnetically collected and air-dried.

### 2.3 Preparation of Dynamic Biocarriers

1 mL of a GQD–MRs dispersion (0.1 mg·mL^–1^ in a 100 mM Tris-HCl buffer solution, pH 7.0) was mixed with 40 nM of the FAM-L probe for 5 min at room temperature to induce supramolecular π-stacking interactions.^28^ The resulting dynamic biocarriers (FAM-L/GQD–MRs) were then properly washed with Tris-HCl buffer by collecting them magnetically. According to the FRET phenomena, the quenching observed at the FL emission band of the FAM-L was indicative of the proper biofunctionalization.^31,51,52^

### 2.4 Optical Assay for DNA Determination

The optical assay for DNA determination was carried out by adding different concentrations (0.05–100 nM) of the DNA target into a fluorescence cell containing a 0.1 mg·mL^–1^ aqueous solution of dynamic biocarriers under H_2_O_2_-induced motion (1% v/v). The mixture was aged for 5 min at room temperature to promote hybridization between the FAM-L probe and the DNA target. The recovery of the FL emission band of the FAM-L probe confirmed the hybridization process. It is important to point out that the “on-the-fly” hybridization assay was optimized by studying different experimental conditions, such as the pH and concentration of the buffered medium and incubation time, as shown in Figure S1.

Finally, experiments with biological samples (urine and human serum) were performed as follows: GQD–MRs were dispersed in the biological fluids (0.1 mg·mL^–1^) and then mixed with a 40 nM FAM-L probe for 5 min at room temperature to induce supramolecular π-stacking interactions. After washing steps by magnetic collection, the motors were incubated with 1 nM DNA target in the corresponding biological fluids and self-propelled by adding 1% H_2_O_2_ as fuel. The experiments were carried out in triplicate (*n* = 3).

### 2.5 Equipment and Procedures

The surface morphology and atomic distribution of GQD–MRs were characterized by using scanning electron microscopy coupled to an energy-dispersive X-ray detector (SEM-EDX, TESCAN LYRA 3 XMH). The charge distribution of the microrobots was determined via ζ-potential by using a Malvern Zetasizer. For the optical measurements, FL measurements were performed by using a Jasco FP-8300 spectrofluorometer at room temperature. The FL emission spectra of GQD–MRs were recorded from 370 to 550 nm using λ_ex_ = 350 nm, while the spectra of dynamic biocarriers were conducted from 500 to 700 nm at the same excitation wavelength, and for the GQD–MRs coupled with the FAM-L probe was recorded at λ_ex_ = 490 nm. In order to avoid the effect of the fuel, optical measurements were carried out by subtracting the background employing the solvent (water containing either 1% v/v H_2_O_2_ or pure water blank experiments). Furthermore, UV–vis absorption spectroscopy (Jasco V-750 spectrophotometer) and a VERTEX 70v FTIR spectrometer were employed to confirm the interactions between GQD–MRs and the biomolecules. A Nikon ECLIPSE TS2R inverted microscope integrated with a Basler digital camera (acA1920–155uc) was utilized to record the autonomous motion behavior of the microrobots. In detail, 10 μL of microrobots were added to a glass slide. Subsequently, single drops of both 1% H_2_O_2_ and 0.1% SDS (v/v) were added to induce the motion while improving the viscosity of the solution thanks to the presence of the SDS surfactant.^53^ To record the motion of the bubble-propelled microrobots, videos were recorded by using NIS Elements Advanced Research software at 25 fps. Fiji software was used to treat the videos in order to calculate the speed and trajectories of the GQD−MRs. Fluorescence microscopy measurements of FAM-L@GQD–MRs were performed using a Nikon Eclipse Ti2 fluorescence microscope employing different excitation wavelengths (green excitation/red emission for GQDs and blue excitation/green emission for FAM-L).

## 3 Results and Discussion

### 3.1 Material Characterization of GQD–MRs

**Figure 1:**
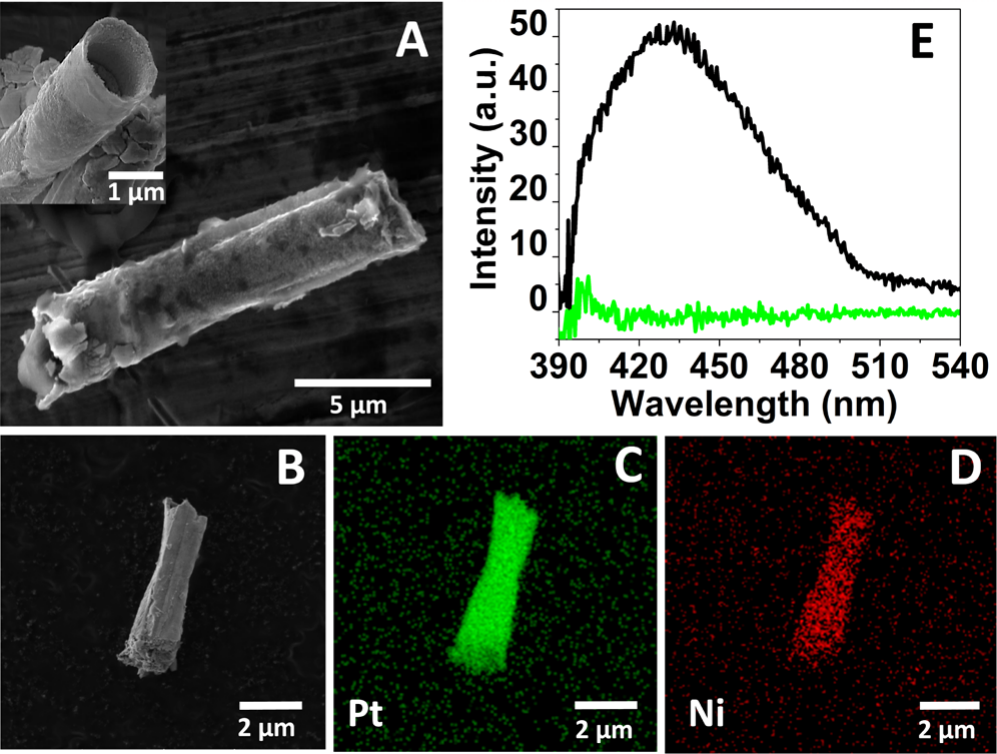
Material characterization of self-propelled GQD–MRs. (A) SEM image (inset: cross-sectional view at higher magnification) and (B) SEM-EDX image of GQD–MRs with its corresponding elemental mapping composition for (C) Pt and (D) Ni. (E) Fluorescence emission spectra of GQD–MRs at λ_ex_ = 350 nm (green line: control emission spectra utilizing the aqueous media without the presence of GQD–MRs).

GQD–MRs were synthesized by following our previously reported membrane-assisted method. To confirm their successful fabrication, material characterization by means of SEM, EDX, and FL analyses was carried out (Figure 1).

Figure 1A displays the lateral SEM image of the GQD–MRs, indicating a microrocket-like structure with a tubular length of around 10 μm and a cross-sectional diameter of around 1 μm (see the inset image). A magnified longitudinal view of the microrobot (Figure 1B) was utilized for the EDX analyses. The elemental mapping composition of the material shown in Figure 1C,D revealed the presence of main elements such as Pt and Ni, respectively. According to these characterization data, results suggest that the self-propelled GQD–MRs made of three layers (GQDs, Ni, and Pt as outer, middle, and inner layers, respectively) were successfully synthesized. Further, the inherent fluorescent features of GQDs^54,55^ were also explored in the resulting GQD–MRs. Figure 1E depicts the emission spectrum of GQD–MRs, with a maximum intensity at 430 nm (excitation light: λ_ex_ = 350 nm). This result clearly confirms that the optical properties of the pristine GQDs were properly transferred to the micromachine.

**Figure 2:**
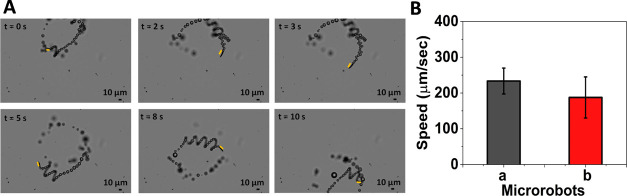
Motion behavior of microrobots. (A) Optical microscopy images showing the trajectory of GQD–MRs at different time intervals (0, 2, 3, 5, 8, and 10 s). Scale bar: 10 μm. The yellow bar is added to guide the eye to follow the microrobot. (B) Histograms depicting the average speed data of GQD–MRs (a) before and (b) after biofunctionalization with the FAM-L probe. Experimental conditions: motion tracking was done in 1% H_2_O_2_ (v/v) for five different microrobots (*n* = 5).

Following this, the bubble-induced self-propulsion behavior of GQD–MRs via H_2_O_2_ decomposition was explored, where the Pt inner layer is the one in charge of catalyzing the fuel.^56−58^ For this aim, the speed of GQD–MRs was monitored by using 1% (v/v) H_2_O_2_. The bubble propulsion of the GQD–MRs was clearly visualized in the micrographs of Figure 2A, with an average speed of as fast as 233 ± 36 μm·s^–1^ (Figure 2B). Taking into account the biotoxicity of H_2_O_2_ at high concentrations, such a low concentration of fuel (1% H_2_O_2_) was chosen in order to not disrupt the following biological purpose: optical “on-the-fly” DNA determination. It is important to highlight that the amount of fuel used in this work is in line with the concentrations of H_2_O_2_ reported by other research groups for biosensing approaches, since in all cases, the experiments are carried out ex vivo.^12,41,59,60^

### 3.2 Exploration of Dynamic Biocarriers Made of GQD–MRs

Having verified the successful synthesis and motion of GQD–MRs, the next step was focused on their exploration toward biological applications. As a proof of principle, the “on-the-fly” DNA determination was considered. For this aim, GQD–MRs were biofunctionalized with the FAM-L probe via π-stacking interactions.

**Figure 3:**
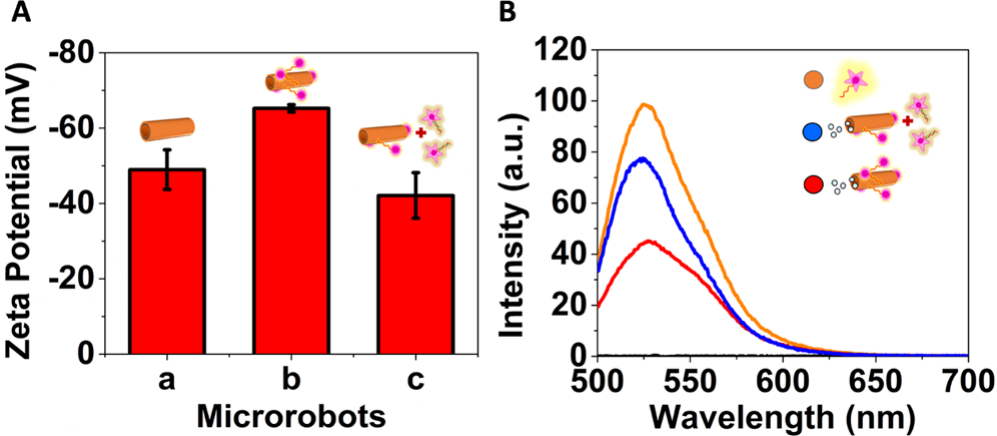
Characterization of microrobots at the different biofunctionalization stages under static conditions. (A) ζ-potential values of GQD–MRs and dynamic biocarriers before and after DNA target interactions. (B) Emission spectra of the FAM-L probe (control, orange line) and dynamic biocarriers before (red line) and after DNA target interactions (blue line). Experimental conditions: λ_ex_ = 490 nm, [FAM-L probe] = 40 nM, hybridization time: 5 min.

First, both GQD–MRs and FAM-L@GQD–MRs were characterized via FTIR spectroscopy. As shown in Figure S2, both spectra present the characteristic absorption bands corresponding to the stretching and bending vibration of the aromatic C–H group at 3385 cm^–1^, C=C stretching at 1637 cm^–1^, C–H aromatic at 2000 cm^–1^, and epoxy stretching vibration of C–O–C groups at 1049 cm^–1^. Unfortunately, after conjugation, the peaks that could be attributed to the amino groups from FAM-L (i.e., located around 654, 1620, and 3310–3350 cm^–1^ corresponding to NH_2_/N–H, N–H, and N–H amines, respectively)^61−63^ clearly overlapped with the weak peaks from the bare GQD–MRs. Consequently, FTIR analysis does not reveal the proper decoration of GQD–MRs with the FAM-L probe. Thus, further characterization was carried out by means of UV–vis spectroscopy. Figure S3 shows the UV–vis spectra of bare GQD–MRs and FAM-L@GQD–MRs (before and after hybridization with the DNA target). While the absorption spectrum of bare GQD–MRs exhibited the typical bands at 240 and 270 nm—assigned to the π–π* transition of C=C in aromatics^64^, a red shift was observed after functionalization with the FAM-L probe, demonstrating a significant change in the optical properties as compared to unmodified GQD–MRs. This fact can be ascribed to the biofunctionalization of GQD–MRs with the FAM-L probe via π-stacking interactions. Importantly, an additional optical change was reached after material hybridization with the DNA target, resulting in a band at 262 nm, suggesting the proper desorption of the FAM-L probe.^65,66^ Additional optical characterization was performed by means of fluorescence microscopy. The optical images shown in Figure S4 evidenced the fluorescence features of both GQDs and the FAM-L probe in the dynamic biocarriers, indicating proper material biofunctionalization. In addition, the speed of the resulting FAM-L@GQD–MR dynamic biocarriers was also monitored, displaying a significant speed decrease from 233 ± 36 to 187 ± 57 μm·s^–1^ when compared with the nonbiofunctionalized counterpart (Figure 2B). This is also an indication that the biomarker might be immobilized on the microrobot surface, since the speed of MRs is influenced by the nature of the surface exposed on the medium. Finally, the charge distribution of GQD–MRs before and after biofunctionalization was recorded by the ζ-potential under various conditions (bare GQD–MRs and FAM-L@GQD–MRs before and after hybridization with the target DNA). As shown in Figure 3A, the ζ-potential value of GQD–MRs decreased from −49 ± 5 to −65 ± 1 mV after biofunctionalization with the FAM-L probe. This ζ-potential decrease is in agreement with the negative phosphodiester backbone of the FAM-L probe.^28^ To demonstrate the suitability of the dynamic biocarriers to interact with a complementary DNA target, they were first incubated with an aliquot of the DNA target (40 nM) for 5 min to promote the DNA hybridization process. As expected, the ζ-potential value was completely recovered after DNA hybridization, yielding a ζ-potential value of −42 ± 6 mV. These data fully demonstrate that the dynamic biocarriers can properly interact with the DNA target, resulting in a release of the biomarker from the GQD–MRs’s walls. It is important to point out that these experiments were run under static conditions (considering the microrobots as passive particles). Further, these results are also in line with the ones obtained by FL measurements (Figure 3B), in which a quenching on the emission band of the FAM-L probe (control) at 527 nm (λ_ex_ = 490) was clearly observed after immobilization on GQD–MRs via π-stacking interactions, while the intensity of the emission band remarkably increased when the dynamic biocarriers were exposed to a fixed concentration of the DNA target driven by the DNA hybridization process. All in all, it is safe to conclude that the FAM-L probe was successfully immobilized on the GQD–MRs’ walls via π-stacking interactions and that the DNA target provided a proper environment to release such interactions after hybridization. Thus, motivated by these promising results obtained under static conditions, the last step was focused on exploiting the devised dynamic biocarriers toward the “on-the-fly” determination of DNA.

### 3.3 Optical “On-the-Fly” DNA Determination Using Dynamic Biocarriers

Once the formation of the dynamic biocarriers and their feasibility toward DNA determination were verified via a DNA hybridization process, the optimization of the “on-the-fly” detection conditions was first studied. This includes optimization of pH, salt concentration, and incubation time (Figure S1). From this optimization study, it can be concluded that the best experimental conditions are pH: 7, salt concentration: 200 nM, and incubation time: 5 min.

As demonstrated by the ζ-potential, UV–vis spectroscopy, and FL analyses, the reported conditions successfully reached the DNA hybridization, which was monitored via the FRET effect by employing different concentrations of the DNA target. During all of the processes, the temperature was fixed to room temperature to make the experiments simpler in the cuvette.

**Figure 4:**
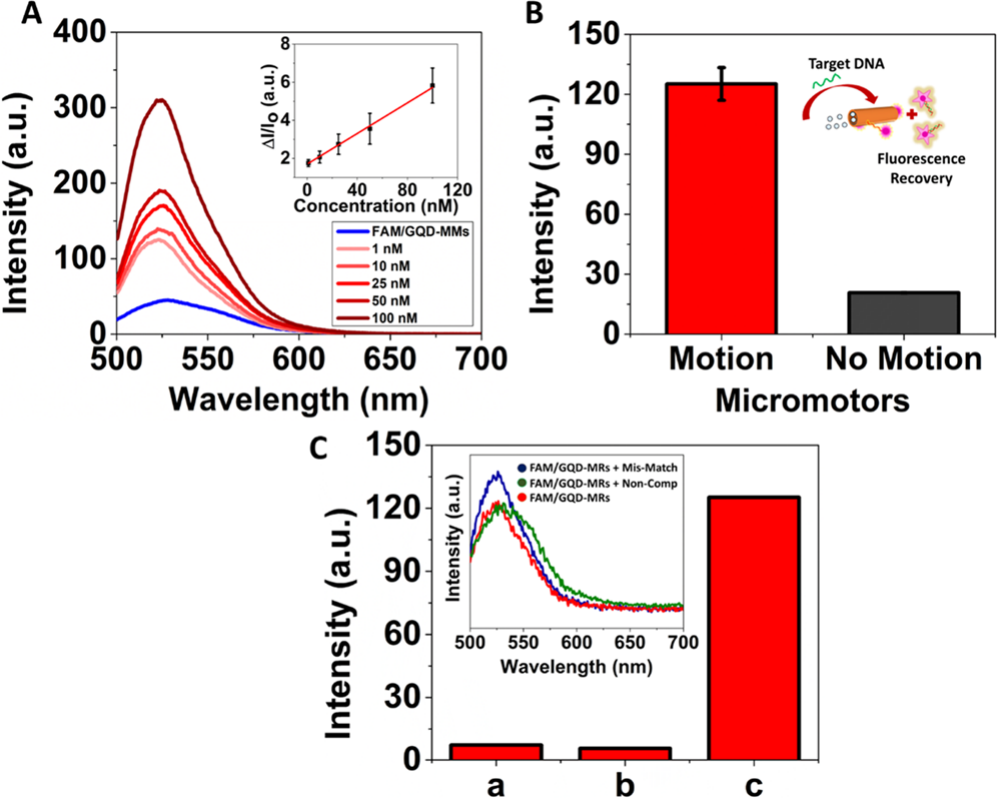
Benefits of exploiting dynamic biocarriers toward the optical “on-the-fly” DNA determination. (A) Emission spectrum of dynamic biocarriers before and after adding different [DNA target], demonstrating how the FL of the FAM-L probe is progressively recovered after “on-the-fly” DNA hybridization (*n* = 3). Inset: calibration plot under motion conditions represented as Δ*I*/*I*_0_ versus [DNA target]. (B) Histogram depicting the FL intensities achieved by the FM/GQD–MRs toward a fixed concentration of the DNA target (1 nM) under motion (1% H_2_O_2_) or nonmotion (no fuel) conditions (inset: schematic representation of microrobots with motion). (C) FL intensities achieved by the dynamic biocarriers toward different targets (1% H_2_O_2_) (i.e., mismatch (a), noncomplementary DNA (b), and DNA target (c); target concentration: 1 nM). Inset: emission spectrum of the dynamic biocarriers (1% H_2_O_2_), i.e., FAM/GQD–MRs (red), FAM/GQD–MRs with noncomplementary DNA (green), and FAM/GQD–MRs with mismatch DNA (blue). Experimental conditions: λ_ex_ = 490 nm, hybridization time: 5 min, concentration (mismatch, noncomplementary DNA): 1 nM.

After these factors were optimized, the last stage was focused on exploring their optical “on-the-fly” biorecognition capabilities. For this aim, a fluorometric assay was run by adding in a quartz cuvette a fixed amount of dynamic biocarriers containing different concentrations of the DNA target in the nM range. Further, the solution was filled with a drop of H_2_O_2_ to reach 1% H_2_O_2_ (v/v) to induce the “on-the-fly” DNA determination. Figure 4 shows the intensity changes on the emission band of FAM-L probe were monitored after 5 min of incubation time. The calibration curve of Figure 4A (inset) is represented as Δ_FL_ = Δ*I*/*I*_0_, where Δ*I* = *I*_*x*_ − *I*_0_ are the fluorescence intensities obtained before (*I*_0_) and after adding different *x* concentrations of the DNA target (*I_x_*). Herein, the optical detection principle relies on monitoring the fluorescence recovery of the quenched FAM-L probe presented in the dynamic biocarriers after incubation with different concentrations of the DNA target (complementary to the FAM-L probe sequence).^67−68,69^ Thus, the addition of the DNA target promotes the hybridization between the FAM-L probe immobilized on the GQD–MRs and the target, leading to double-stranded DNA (ds-DNA) formation. Since the resulting ds-DNA has a poor binding affinity to the GQD–MRs,^70,71^ it is released from the surface of the dynamic biocarriers, resulting in a fluorescence intensity recovery. Hence, the changes in the fluorescence intensity band of the FAM-L probe with regard to different concentrations of the DNA target (optical readouts) were the key to the “on-the-fly” DNA determination.

As shown in Figure S5, the “on-the-fly” DNA determination study was conducted via FL analyses, where the emission band intensity increases with increasing concentration of the DNA target ranging from 0.05 to 100 nM, whereas a linear range was obtained from 1 to 100 nM (Figure 4A). Figure 4A (inset) displays a linear plot of [DNA target] versus Δ_FL_ with its corresponding error bars (*n* = 3). An excellent calibration curve—Δ_FL_ = 1.70 + 0.04 [DNA target] (nM), *R*^2^ = 0.99—in the 1.0–100 nM range was yielded, with a detection limit as low as 0.05 nM. Importantly, a control calibration plot under static conditions (without H_2_O_2_-induced motion) was also run in order to demonstrate the benefits of the dynamic biocarriers (Figure S6). As shown in Figure 4B, the sensitivity of the method was 3 times enhanced under motion conditions. In addition, while the dynamic biocarriers obtained an excellent linear trend with increasing the [DNA target] through the “on-the-fly” analysis, a nonlinear trend was observed during the static control (Figure S7). Principally, the continuous motion of the microrobots enhanced mass transport within the solution. Further, due to the propulsion of the microrobots, the rate of diffusion increases, which amplifies the homogeneous dispersion of desired materials. Thus, this enabled them to enhance the efficiency, velocity, and yield of their processes. Hence, it demonstrates the pivotal role of “on-the-fly” analyses to rapidly interact and/or intimate with the target of interest, making it possible to reach better sensitivities. Compared to the state-of-the-art DNA-based MRs utilized so far for the optical “on-the-fly” determination of DNA (see Table S1),^39,40,42,72^ to the best of our knowledge, this supposes the first GQD-based MRs for optically monitoring DNA, presenting one of the lowest detection limits (0.05 nM vs 10–1300 nM). Only the work carried out by Wu et al. surpassed the presented GQD-based MRs, where a detection limit of 0.01 nM was yielded using Au–Pt bimetallic nanomotors.^40^

The selectivity of the dynamic biocarriers toward the complementary DNA target was evaluated by exploring alternative DNA targets, such as mismatch and noncomplementary DNA sequences. As shown in Figure 4C, a significant decrease in the emission band intensity was observed after the mismatch DNA hybridized with the FAM-L probe anchored to the GQD–MRs. Nonetheless, such intensity was 15 times lower than the one achieved by the complementary DNA target. Otherwise, almost no fluorescence response was evidenced when the dynamic biocarriers were exposed to the noncomplementary DNA target. This suggests that the devised GQD-based dynamic biocarriers preferably interact with the complementary DNA target, validating the selectivity of the microrobots.

Finally, in order to validate its applicability in biological samples, the “on-the-fly” determination of DNA was also monitored by employing biological fluids (urine and human plasma). To perform this study, a 1 nM DNA target was spiked to urine and human plasma samples in the presence of dynamic biocarriers under motion conditions (fuel concentration: 1% H_2_O_2_), and the recovered concentration was optically quantified per triplicate (*n =* 3) by extrapolation in the calibration curve from Figure 4A (inset). The average concentrations of the DNA target found in both urine and human plasma were 1.27 ± 0.34 and 1.31 ± 0.07 nM, respectively. These results evidenced a nonsignificant interfering effect from the complex matrices, demonstrating that the dynamic biocarriers can also be utilized for the “on-the-fly” monitoring of DNA in biological fluids.

## 4 Conclusions

Herein, a facile and cost-effective fabrication of dynamic biocarriers made of GQD-based self-propelled microrobots carrying a biological marker is presented for their exploration as unconventional optical platforms for enhancing analytical assays via “on-the-fly” interactions. As a proof of concept, the “on-the-fly” determination of DNA has been considered by immobilizing a DNA probe (namely, FAM-L probe) via supramolecular π–π interactions, obtaining excellent detection limits and selectivity when compared with nonspecific/mismatch DNA targets. Remarkably, this method was 3 times enhanced when the microrobots were in motion mode; in other words, when the biocarriers were in dynamic mode by taking advantage of the fuel (1% H_2_O_2_). In addition, the feasibility of the developed dynamic biocarriers was also explored by spiking the DNA target in two different biological fluids (i.e., urine and plasma), demonstrating, in both cases, promising recoveries. Consequently, the preparation of hybrid MRs by combining different materials exhibiting different features (i.e., GQD with fluorescence and π-stacking interactions, Pt with self-propelling capabilities, and Ni responding to an external magnetic field) provides multifunctional microrobots capable of performing analytical tasks in a rapid, sensitive, and selective way. This work opens the way for rapid DNA optical assays, which are dramatically enhanced by the presence of micromachines.

## Supporting Information Available

Figure S1. (A) Hybridization time optimization study (target DNA hybridization (1 nM) with FAM-L probe (40 nM)) (red) GQDs-MRs in motion by using1% H2O2 (black) GQDs-MRs without motion. (B) Effect of pH study (5, 6, 7, 8) on DNA hybridization using GQD–MRs. While pH 5 exhibited poor signals due to partial protonation of ssDNA’s phosphate backbone, resulting in weak hybridization, at pH 6 protonation and deprotonation led to a slightly higher signal; Figure S2. The FTIR spectra of the bare GQD–MRs (black), FAM-L@GQD–MRs (red); Figure S3. The UV-vis absorption spectrum of the bare GQD–MRs (blue), FAM-L@GQD–MRs before (red) and after hybridization with the target DNA (green); Figure S4. (a) is depicting the control of the GQD–MR in the bright field mode. (b) fluorescence due to the GQDs embedded on the surface of the microrobots. (c) fluorescence of FAM-L probe present on the surface of the GQD–MRs. (d) is depicting the multichannel image of the GQD–MR with DNA probe on its surface; Figure S5. Effect of the different concentrations (0.05, 0.10, 0.5, 1, 10, 25, 50, 100 nM) of the target DNA on the fluorescence emission spectra of the GQDs-MRs (with motion); Figure S6. Effect of the different concentrations (1, 10, 25, 100 nM) of the target DNA on the fluorescence emission spectra of the GQDs-MRs (no motion); Figure S7. Comparison between the fluorescence intensity of the FAM-L probe (40 nM) hybridized with the target DNA (variable concentration); Table S1. Table showing different microrobots exploited towards the optical “on-the-fly” determination of DNA (PDF)
