# Supplementary material for: Quantum Material-Based Self-Propelled Microrobots for the Optical “On-the-Fly” Monitoring of DNA
Source: ACS Appl Mater Interfaces. 2023 Dec 11;15(50):58548–55. doi: 10.1021/acsami.3c09920 (PMC10750807; doi:10.1021/acsami.3c09920)
Supplement: Supplementary file 1 — am3c09920_si_001.pdf [file am3c09920_si_001.pdf]

Supporting Information

Quantum materials-based self-propelled microrobots for optical 'on-the-fly' monitoring of DNA

Jyoti,[a] Jose Muñoz,[a] Martin Pumera[a,b,c*]

[a] Future Energy and Innovation Laboratory, Central European Institute of Technology, Brno University of Technology (CEITEC-BUT), 61200 Brno, Czech Republic

[b] Faculty of Electrical Engineering and Computer Science, VSB - Technical University of Ostrava, 17. listopadu 2172/15, 70800 Ostrava, Czech Republic

[c] Department of Medical Research, China Medical University Hospital, China Medical University, No. 91 Hsueh-Shih Road, Taichung 4040

Email Address:

Martin.pumera@ceitec.vutbr.cz

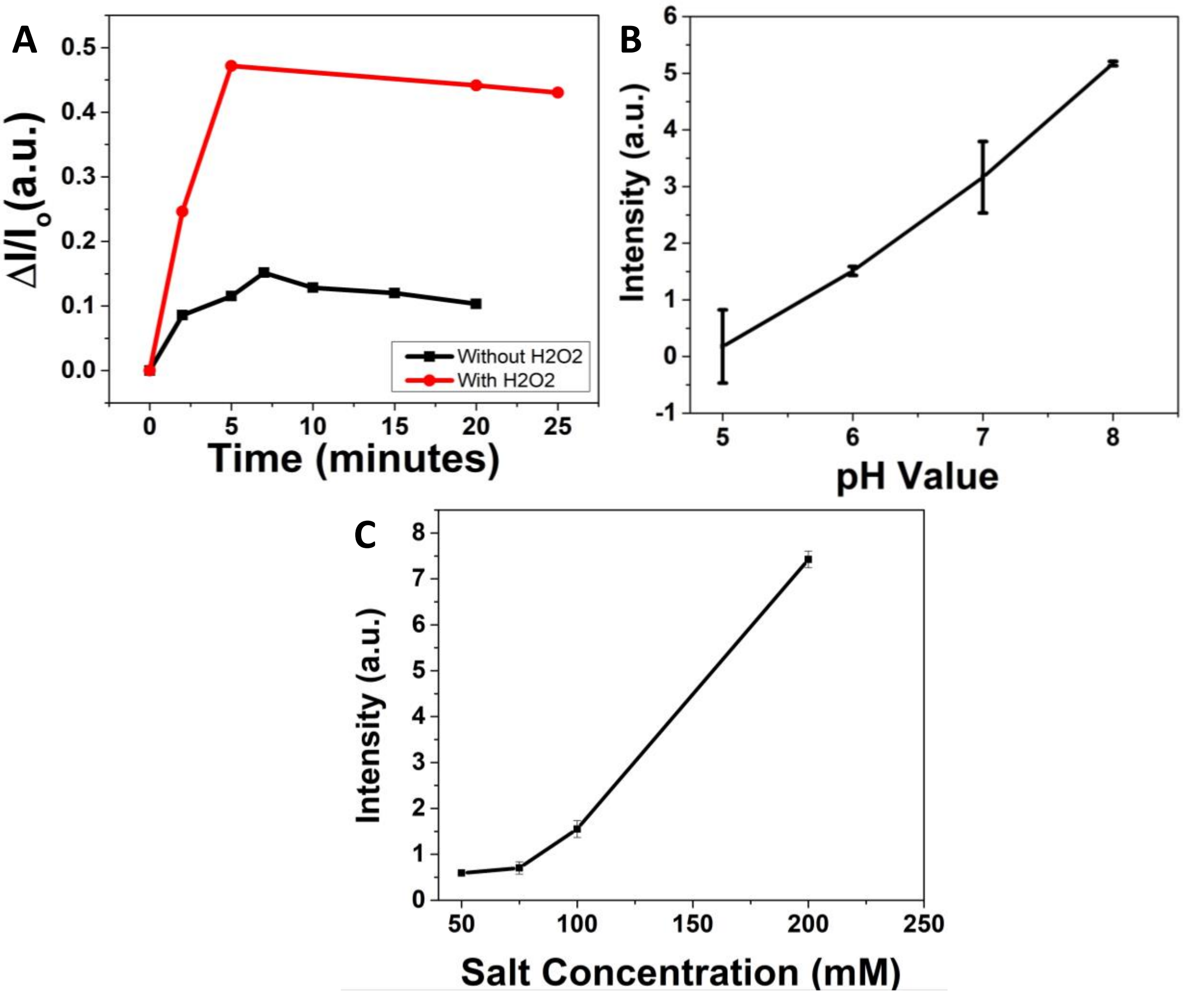

Figure S1: Optimization of the hybridization conditions **(A)** Hybridization time optimization study (target DNA hybridization (1 nM) with FAM-L probe (40 nM)) **(red)** GQD–MRs in motion by using 1% H_2O_2 **(black)** GQD–MRs without motion. During the time optimization study, the fluorescence intensity increased with the increase in time up to 7 minutes (in case no H_2O_2) whereas up to 5 minutes (in case H_2O_2). **(B)** Effect of pH study (5, 6, 7, 8) on DNA hybridization using GQD–MRs. While pH 5 exhibited poor signals due to partial protonation of ssDNA's phosphate backbone, resulting in weak hybridization, at pH 6 protonation and deprotonation led to a slightly higher signal. A significant signal increase occurred at pH 7, peaking at pH 8, indicating pH strongly influences hybridization efficiency.[1–3] **(C)** Effect of the salt concentration (50, 75, 100, 200 mM) on the DNA hybridization process. Increasing Tris-HCl concentration (50 to 200 mM) enhanced the peak, possibly due to Tris molecules forming hydrogen bonds with DNA backbones and binding to solid surfaces via -$NH3^+$ groups. Additionally, -$NH3^+$ groups attached to DNA phosphate groups, while -OH groups formed hydrogen bonds with the adsorbent surface, contributing to the observed increase.[4,5] Conditions: Excitation wavelength 490 nm.

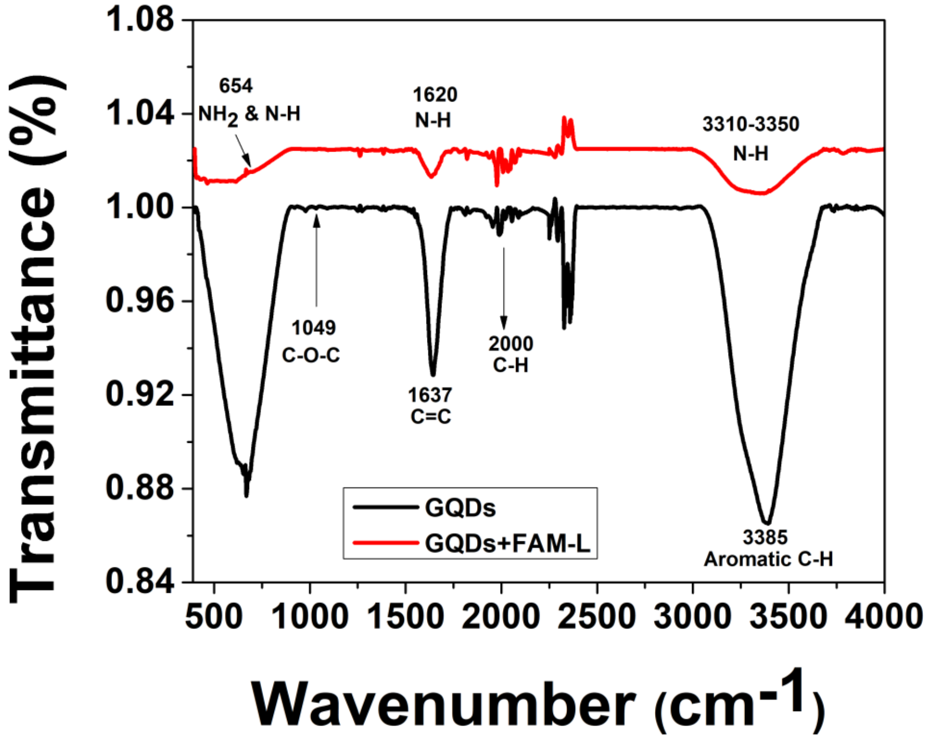

Figure S2: FTIR characterization of the bare GQD–MRs (black), FAM-L@GQD–MRs (red).

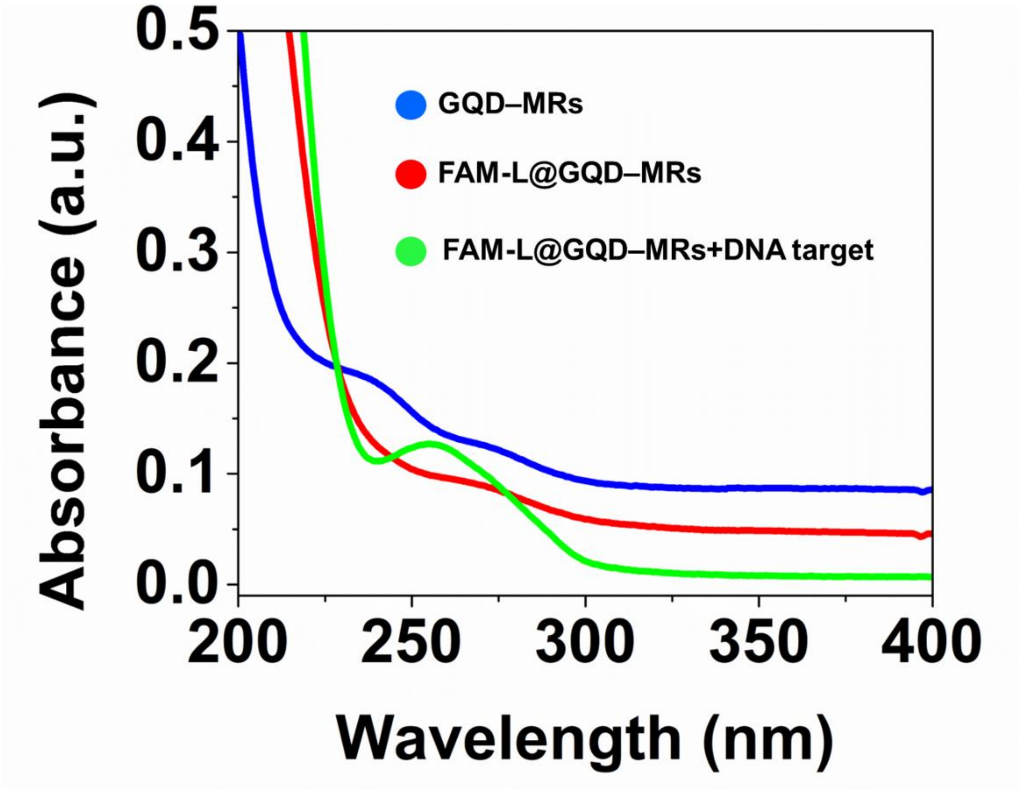

Figure S3: UV-vis characterization: the UV-vis absorption spectrum of the bare GQD–MRs (blue), FAM-L@GQD–MRs before (red) and after hybridization with the target DNA (green).

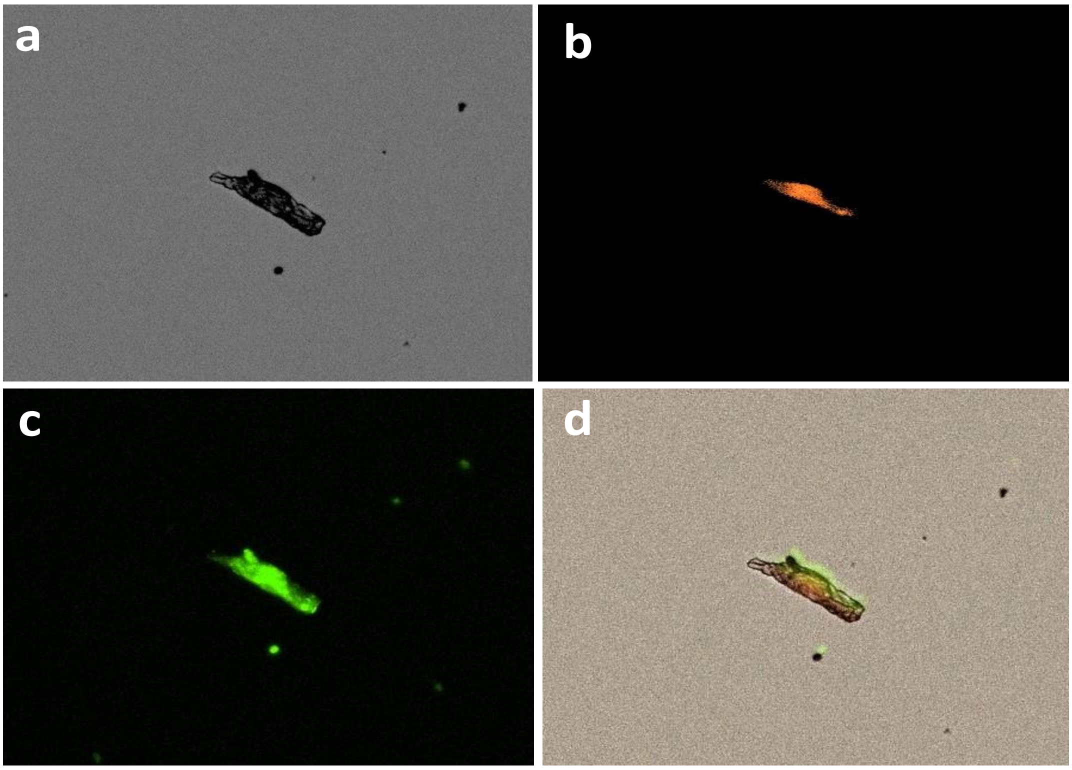

Figure S4: Fluorescence characterization (a) is depicting the control of the GQD–MR in the bright field mode. (b) fluorescence due to the GQDs embedded on the surface of the microrobots. (c) fluorescence of FAM-L probe present on the surface of the GQD–MRs. (d) is depicting the multichannel image of the GQD–MR with DNA probe on its surface.

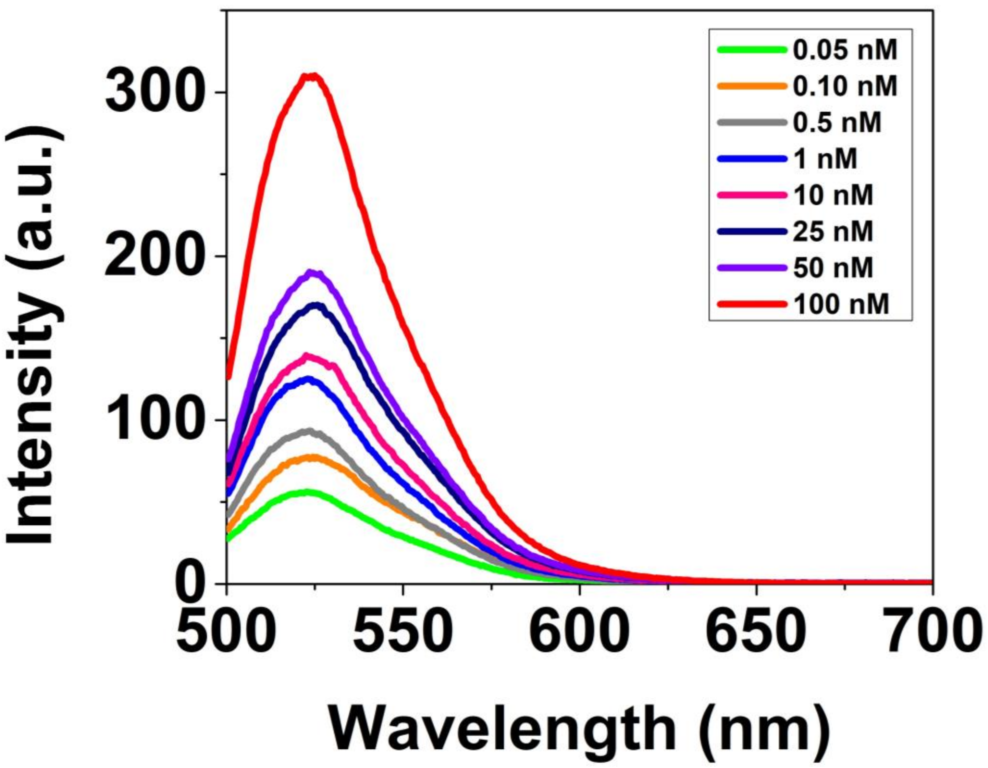

Figure S5: Fluorometric “on-the-fly” assay at different target concentrations (0.05, 0.10, 0.5, 1, 10, 25, 50, 100 nM) on the fluorescence emission spectra of the GQD–MRs (with motion). Conditions: Excitation wavelength 490 nm, FAM-L probe concentration 40 nM, hybridization time 5 minutes.

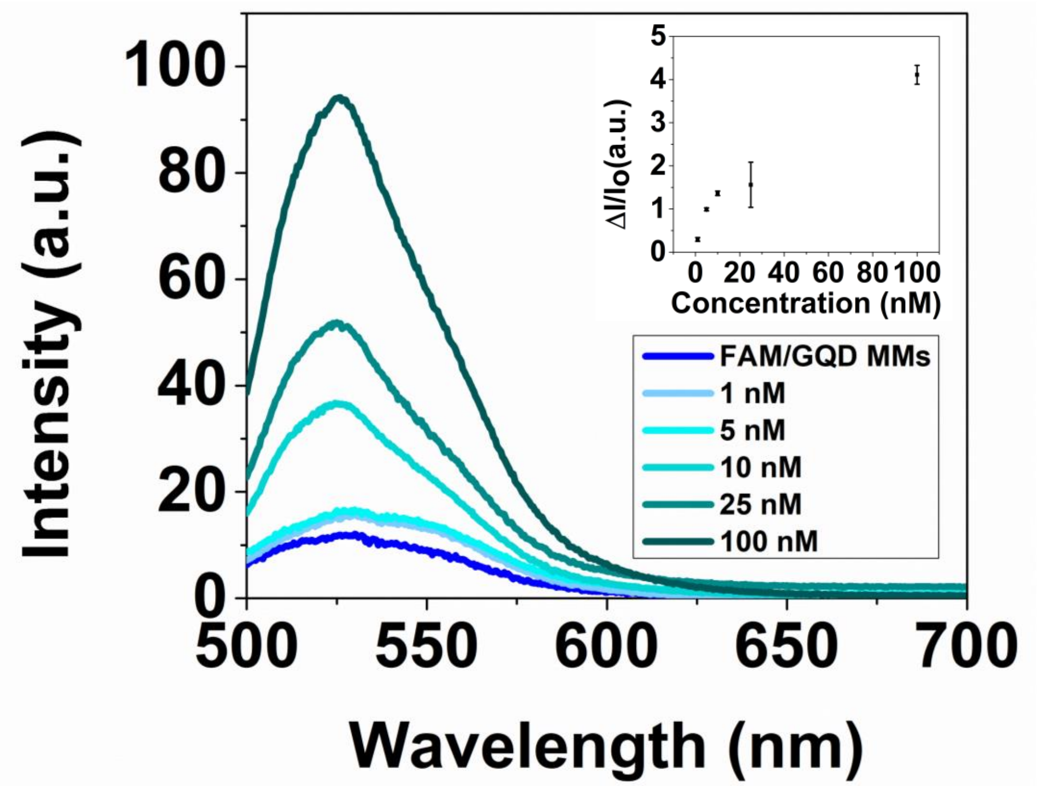

Figure S6. Fluorometric assay at different target concentrations (1, 10, 25, 100 nM) under static conditions**.** An inset shows the concentration calibration curve for the target DNA hybridization based on the performance of GQD–MRs in the absence of motion. Conditions: Excitation wavelength 490 nm, FAM-L probe concentration 40 nM, hybridization time 5 minutes.

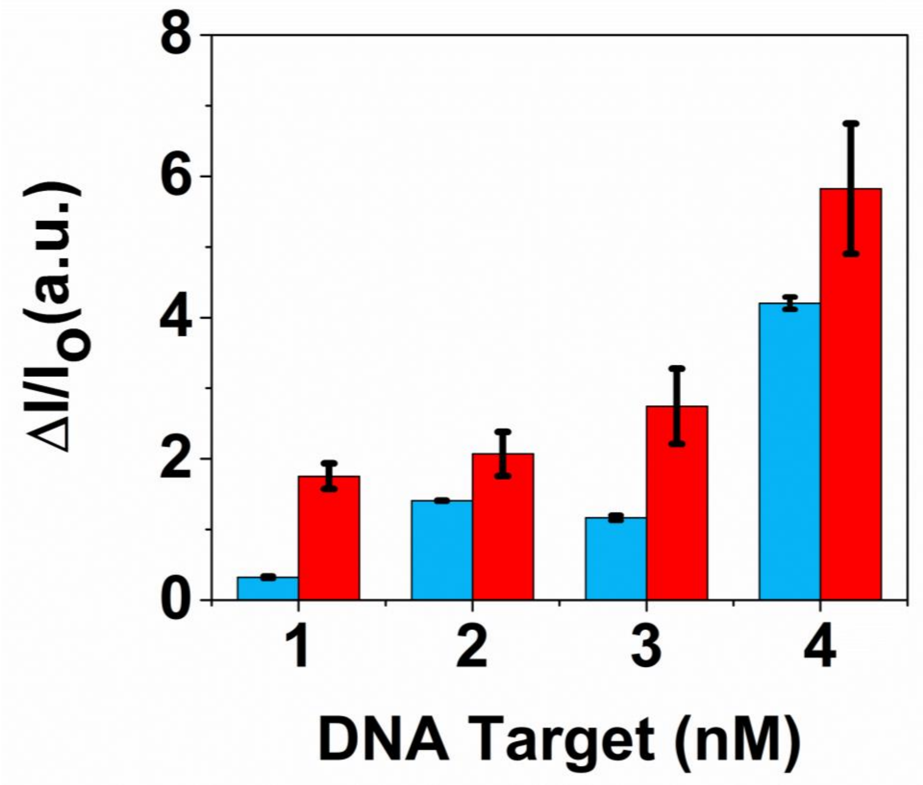

Figure S7. Fluorometric assay comparison motion vs. no motion. **Red** depicting the fluorescence response of the FAM-L probe, when GQD–MRs were in motion while **blue** represents the micromotors without motion. Conditions: Excitation wavelength 490 nm, FAM-L probe concentration 40 nM, hybridization time 5 minutes.

Table S1: Table comparing optical "on-the-fly" determination of DNA using microrobots.

Type of Micromotors	Target detected	Detection Range	Environment	Detection Limit	Reference
Catalytic Nanomotors (Au-Pt bimetallic nanowire)	DNA sensing	0.01 -100 nM	In-vitro	0.01 nM	[6]
Enzyme-powered micromotor	DNA detection	10 nM to 1000 nM	In-vitro	10 nM	[7]
Catalytic, magnetic and hybrid micromotors	Gastric cancer biomarker detection	1000 to 10000 nM	In-vitro	1300 nM	[8]
Helical hydrogel micromotor	Tumor DNA detection	2500–25,000 nM	In-vitro	250 nM	[9]
GQD-MRs-based dynamic biocarriers	DNA detection	1–100 nM	In-vitro	0.05 nM	Present work

References:

(1) Zhang, J.; Lang, H. P.; Yoshikawa, G.; Gerber, C. Optimization of DNA Hybridization Efficiency by PH-Driven Nanomechanical Bending. *Langmuir* **2012**. https://doi.org/10.1021/la205066h.

(2) Lee, J.; Kim, J.; Kim, S.; Min, D. H. Biosensors Based on Graphene Oxide and Its Biomedical Application. *Advanced Drug Delivery Reviews*. 2016. https://doi.org/10.1016/j.addr.2016.06.001.

(3) Rashid, J. I. A.; Yusof, N. A. The Strategies of DNA Immobilization and Hybridization Detection Mechanism in the Construction of Electrochemical DNA Sensor: A Review. *Sensing and Bio-Sensing Research* **2017**, *16*, 19–31. https://doi.org/10.1016/j.sbsr.2017.09.001.

(4) Saeki, K.; Kunito, T.; Sakai, M. Effect of Tris-HCl Buffer on DNA Adsorption by a Variety of Soil Constituents. *Microbes and Environments* **2011**. https://doi.org/10.1264/jsme2.ME10172.

(5) Jeong, S.; Pinals, R. L.; Dharmadhikari, B.; Song, H.; Kalluri, A.; Debnath, D.; Wu, Q.; Ham, M. H.; Patra, P.; Landry, M. P. Graphene Quantum Dot Oxidation Governs

Noncovalent Biopolymer Adsorption. *Scientific Reports* **2020**. https://doi.org/10.1038/s41598-020-63769-z.

(6) Wu, J.; Balasubramanian, S.; Kagan, D.; Manesh, K. M.; Campuzano, S.; Wang, J. Motion-Based DNA Detection Using Catalytic Nanomotors. *Nature Communications* **2010**. https://doi.org/10.1038/ncomms1035.

(7) Fu, S.; Zhang, X.; Xie, Y.; Wu, J.; Ju, H. An Efficient Enzyme-Powered Micromotor Device Fabricated by Cyclic Alternate Hybridization Assembly for DNA Detection. *Nanoscale* **2017**. https://doi.org/10.1039/c7nr01168g.

(8) Báez, D. F.; Ramos, G.; Corvalán, A.; Cordero, M. L.; Bollo, S.; Kogan, M. J. Effects of Preparation on Catalytic, Magnetic and Hybrid Micromotors on Their Functional Features and Application in Gastric Cancer Biomarker Detection. *Sensors and Actuators, B: Chemical* **2020**. https://doi.org/10.1016/j.snb.2020.127843.

(9) Qin, F.; Wu, J.; Fu, D.; Feng, Y.; Gao, C.; Xie, D.; Fu, S.; Liu, S.; Wilson, D. A.; Peng, F. Magnetically Driven Helical Hydrogel Micromotor for Tumor DNA Detection. *Applied Materials Today* **2022**. https://doi.org/10.1016/j.apmt.2022.101456.
